# Prevention of falls in hospitalized patients—evaluation of the effectiveness of a monitoring system (Verso Vision) developed with artificial intelligence

**DOI:** 10.3389/fdgth.2025.1548209

**Published:** 2025-04-16

**Authors:** Corrado Gervasi, Erik Perego, Francesca Galli, Valter Torri, Massimo Castoldi, Emilio Bombardieri

**Affiliations:** ^1^ Nursing Service, Humanitas Gavazzeni, Bergamo, Italy; ^2^ Medical Direction, Humanitas Gavazzeni, Bergamo, Italy; ^3^ Methodology Clinical Research Laboratory, Istituto di Ricerche Farmacologiche Mario Negri, IRCCS, Milano, Italy; ^4^ Department of Clinical Oncology, Istituto di Ricerche Farmacologiche Mario Negri, IRCCS, Milano, Italy; ^5^ Scientific Direction, Humanitas Gavazzeni, Bergamo, Italy

**Keywords:** accidental falls, artificial intelligence, hospitalization, prevention, remote monitoring

## Abstract

**Introduction:**

The prevention of accidental falls in hospital is an important aspect of a healthcare management strategy, since they represent a relevant socio-economic problem. The Verso Vision System (VS) is an artificial intelligence-based system for accidental fall prevention and management, which uses computer vision algorithms to monitor environments and people in real time.

**Methods:**

The efficacy of VS monitoring in terms of reduction of accidentals falls was retrospectively evaluated in a group of 362 hospitalized patients at Humanitas Gavazzeni Hospital.

**Results:**

Of the 362 patients included in the analysis, 580 statistical units, 228 monitored with VS and 355 without VS were obtained splitting the observation of each patient based on the presence of VS monitoring and the Stratify score. The mean age of the 362 patients was 75.3 years and 150 were females (41.4%). The crude incidence rates per 1,000 person-time was 2.85 (95% CI 0.92–6.63, 5 accidental falls) and 6.65 (95% CI 3.72–10.96, 15 accidental falls) in the monitored with VS and unmonitored groups, respectively. At multivariable Poisson regression model, a statistically significant reduction of the risk of accidental falls was found in the monitored group compared to the unmonitored group [incidence rate ratio (IRR) 0.21, 95% CI 0.12–0.38, *p* < 0.0001]. The positive impact was supported by sensitivity analysis (IRR 0.22, 95% CI 0.13–0.35, *p* < 0.0001).

**Conclusion:**

This analysis suggests that the VS can reduce the number of accidental falls in hospitalized patients. Nonetheless, further prospective analyses are needed to confirmed the efficacy of the VS.

## 1 Introduction

The term accidental fall indicates a sudden, unintentional and unexpected downward displacement from an orthostatic or clinostatic position (1). Globally, around 20 million years of life are lost annually due to accidental falls with substantial social and economic consequences. In economically developed countries, healthcare costs related to falls account for about 1% of all health care costs (2).

The incidence of falls is higher among the elderly who reside in nursing homes or are cared for in hospital. This suggests that risk factors may differ between places and settings, which may have relevance for preventive strategies (3). In fact, falls in hospital are among the most common adverse events reported by healthcare professionals.

Prevention of accidental falls in hospitalized patients is very problematic. In Humanitas Gavazzeni hospital, various approaches, recommended by the literature, have been implemented and have progressively entered into daily routine, such as assessment of fall risk using validated scales; patient and caregiver education with written material; training healthcare personnel on the topic of falls and clinical audits to discuss falls in the hospital; adoption of an assisted environment; limitation of use of sedative drugs and improvement of nutritional status. The literature also recommends the use of alarm movement sensors and to create a statistical model of falls after adequate data analysis and use of artificial intelligence (AI) (4–8).

Besides these systems, it seemed clear that the possibility to have a tool that is able to exert continuous remote control and induce rapid intervention by nursing staff could be a very interesting solution. Therefore, the availability of the instrument Verso Vision System (VS) capable of 24-hour monitoring of at-risk movements and/or falls and transmitting alarm signals to nurses in order to stimulate prompt intervention, based on software implemented with AI, gave us the opportunity to study its effectiveness and try to understand if the system is a reliable strategy.

This paper describes a pragmatic retrospective study conducted in our hospital to evaluate the feasibility of implementing VS technology and to provide preliminary insights into its effectiveness in reducing the number of accidental falls during hospital stay.

## 2 Patients and methods

This retrospective analysis included all consecutive patients admitted to the Medicine and Oncology wards of Humanitas Gavazzeni hospital between November 15, 2023 and February 15, 2024. Approval to conduct this study was obtained from Local Ethics Committee. Upon admission to the ward, the patient underwent nursing assessments, which included a fall risk assessment using the Stratify scale (evaluation of previous falls since admission, presence of agitation or confusion, impaired daily functioning due to visual impairment, need for frequent toileting, and requirement for physical assistance with transfers or mobility), the Barthel Index for physical validity and autonomy, and a general evaluation of critical factors (such as age, patient compliance, drug therapy, comorbidities) (9, 10). Consequently, at the time of hospitalization, patients were assigned to either a monitored or an unmonitored bed based on their fall risk (Stratify Index) and the presence of additional factors such as comorbidities and specific medication use.

### 2.1 System description

**Figure 1:**
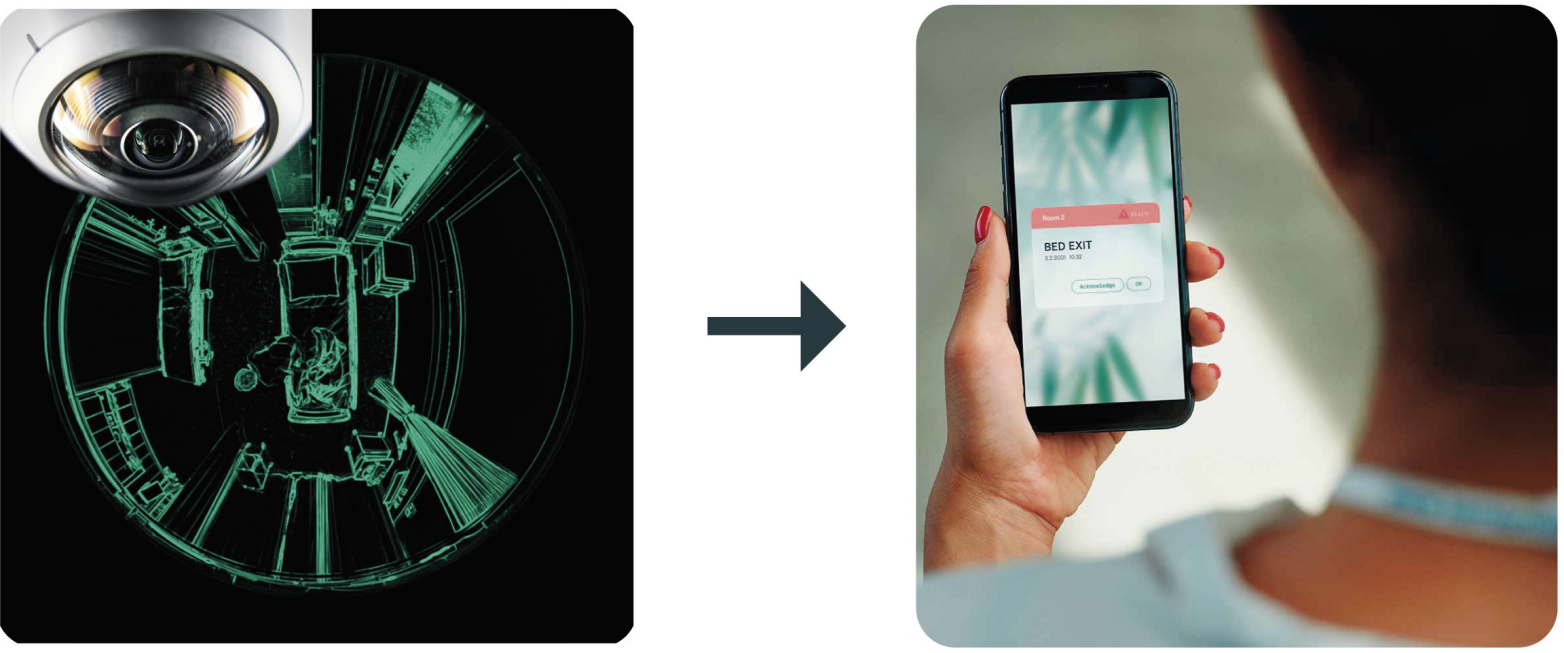
On the left, a fixed camera monitors patients within the room's field of view and detects their movements. Anonymized data are processed by an AI system to analyze potentially risky situations and falls that may require notifying the nursing personnel. On the right, a remote mobile device receives and displays alerts sent to the nursing personnel.

The tool we analyzed to prevent accidental falls is the VS (11). This artificial intelligence-based system for accidental fall prevention and management uses computer vision algorithms to monitor environments and people in real time. In detail, cameras are installed in inpatient rooms to capture videos of the environment and patients. Images are analyzed in order to have information about body position, movements, and precarious conditions while maintaining the anonymity of the individual patient. AI algorithms analyze images to identify risky situations and falls (Table 1). The system guarantees continuous image analysis and critical situation recognition 24 h a day that is transmitted to the nurses in their wards (Figure 1). If a risky situation is detected, the system sends alerts to caregivers via smartphones, enabling timely intervention. Prevention of falls was achieved through prompt intervention by nurses as soon as they received the alarm on the tablet. The immediate action consisted in assisting the patient, and getting them into a safe position. The data collected by the system is used to continuously improve algorithms and optimize prevention strategies, making the system increasingly effective. In this way, the system not only helps prevent falls, but also helps manage them in a timely manner.

**Table 1:** Patient's conditions detected and transmitted as alerts by the VS technology.

Situations detected by VS alarm	Description
Attempt to leave bed	An alert is triggered when the patient initiates an attempt to leave the bed by moving their legs out of it
Bed exit	An alert is triggered when the patient walks a few steps away from the bed
Toilet timer exceeded	When the patient enters the toilet, a timer preset by the nurse is activated in the system. An alert is triggered if the patient remains inside beyond the preset time
Room exit	An alert is triggered when the patient leaves the room
Falls detected	An alert is triggered when a fall is detected by the system

### 2.2 System installation

Twenty beds in 10 rooms of the Medicine and Oncology wards of Humanitas Gavazzeni hospital were selected to be monitored by the VS. The nursing staff involved in the study underwent an appropriate training period for theoretical knowledge of the system and management via smartphones.

### 2.3 Statistical methods

The main objective of this retrospective analysis was to explore the impact of VS technology in terms of reduction of accidental falls during hospitalization.

During hospitalization, patients could be either monitored and not monitored by the VS system. Moreover, the Stratify score evaluation could be repeated based on clinical decisions. Therefore, the statistical unit was obtained splitting the follow-up of each patient based on the presence of VS monitoring and according to changes to the Stratify score.

Univariable and multivariable Poisson regression models were used to investigate the associations of VS and demographic and clinical characteristics and the number of accidental falls. To control for the different durations of observation, the natural logarithm of the length of observation was set as the offset variable. To control for overdispersion, deviance was used as a scale parameter. The Akaike information criterion was used to select the best multivariable model including the application of VS technology and the Stratify score. Results are provided as exponents of model coefficients and 95% confidence intervals (95% CIs). The exponents of the intercept are equal to the incidence rate of population defined by the reference categories of the variables included. The exponents of coefficients are equal to the incidence rate ratio (IRR) with respect to the reference category.

To assess the robustness of results, a sensitivity analysis was performed by applying a propensity score weighting to obtain two balanced group of patients, those monitored and those not monitored with VS technology. Subsequently, the same methods described above were applied to the weighted observations.

Continuous variables were summarized by mean, standard deviation (SD), first quartile (Q1), median, third quartile (Q3) and ranges (minimum and maximum). Categorical variables were summarized by frequency and proportion of each patient in each category. To compare groups, a generalized linear mixed model was used to control for the correlation between observations.

All analyses were performed using the SAS software, version 9.4 (SAS Institute). *P*-values <0.05 were considered statistically significant.

## 3 Results

Overall, 362 patients were admitted to the Medicine and Oncology wards of Humanitas Gavazzeni hospital.

The mean age was 75.3 years (SD 14.2) and there were 150 (41.4%) women. The median length of hospitalization was 8 days (Q1–Q3 3–14).

During hospitalization 193 (53.3%) patients were not monitored with VS, 84 (23.2%) were always monitored, whereas 85 (23.5%) patients were monitored with VS during a partial period of the hospital admission. Moreover, the Stratify score assessed at admission changed for 107 (29.6%) patients. Therefore, the analysis was performed on 580 observations (considered as statistical units), 352 in the group without VS and 228 in the group with VS.

Demographic and clinical characteristics are provided in Table 2. No differences were seen in age and sex between groups. As expected, a statistically significant difference was found between groups in terms of Stratify score with a higher proportion of patients with a score ≥2 among cases with VS monitoring (46.9% vs. 17.9%, *p* < 0.0001). Moreover, a higher proportion of cases in the VS group had cardiac abnormalities (46.2% vs. 33.8%, *p* = 0.0476) and were hospitalized due to symptoms, signs and ill-defined conditions (15.4% vs. 8.8%, *p* = 0.0108). No differences were found in terms of Barthel index and presence of psychiatric or thorax abnormalities.

**Table 2:** Demographic and clinical characteristics of the samples.

Variable	No VS	VS	Overall	*p*-value
	*N* = 352	*N* = 228	*N* = 580	
Age (years)				0.4937
Mean (SD)	75.8 (14.5)	76.9 (12.8)	76.3 (13.8)	
Median (Q1–Q3)	79.7 (67.7–86.4)	77.5 (69.8–86.8)	78.4 (69.1–86.7)	
Min–Max	23.8–100.9	27.9–100.9	23.8–100.9	
Age—*n* (%)				0.0943
<70 years	102 (29.0)	58 (25.4)	160 (27.6)	
70–89 years	210 (59.7)	131 (57.5)	341 (58.8)	
≥90 years	40 (11.4)	39 (17.1)	79 (13.6)	
Female sex—*n* (%)	143 (40.6)	100 (43.9)	243 (41.9)	0.5031
Stratify score—*n* (%)				<0.0001*
0	177 (50.3)	68 (29.8)	245 (42.2)	
1	112 (31.8)	53 (23.2)	165 (28.4)	
2	51 (14.5)	81 (35.5)	132 (22.8)	
3	11 (3.1)	21 (9.2)	32 (5.5)	
4	1 (0.3)	4 (1.8)	5 (0.9)	
5	0 (0.0)	1 (0.4)	1 (0.2)	
Barthel index—*n* (%)				0.1454
0–20	104 (30.1)	81 (35.8)	185 (32.3)	
25–65	112 (32.4)	77 (34.1)	189 (33.0)	
70–100	130 (37.6)	68 (30.1)	198 (34.6)	
Missing	6	2	8	
Psychiatric abnormalities—*n* (%)	155 (44.8)	113 (50.7)	268 (47.1)	0.3922
Missing	6	5	11	
Thorax abnormalities—*n* (%)	213 (61.6)	132 (59.2)	345 (60.6)	0.8138
Missing	6	5	11	
Heart abnormalities—*n* (%)	117 (33.8)	103 (46.2)	220 (38.7)	0.0476
Missing	6	5	11	
Reason for admission to hospital				
Diseases of the respiratory system—*n* (%)	112 (31.8)	57 (25.0)	169 (29.1)	0.0816
Diseases of the circulatory system—*n* (%)	42 (11.9)	31 (13.6)	73 (12.6)	0.5169
Symptoms, signs, and ill-defined conditions—*n* (%)	31 (8.8)	35 (15.4)	66 (11.4)	0.0108
Diseases of the digestive system—*n* (%)	35 (9.9)	19 (8.3)	54 (9.3)	0.5001
Infectious and parasitic diseases—*n* (%)	33 (9.4)	19 (8.3)	52 (9.0)	0.6280
Encounter for other and unspecified procedures and follow-up care—*n* (%)	33 (9.4)	13 (5.7)	46 (7.9)	0.0909
Injury and poisoning—*n* (%)	13 (3.7)	16 (7.0)	29 (5.0)	0.0940
Other—*n* (%)	53 (15.1)	38 (16.7)	91 (15.7)	0.5709
Length of observation (days)				0.0124
Mean (SD)	6.4 (5.7)	7.7 (6.5)	6.9 (6.1)	
Median (Q1–Q3)	5.0 (3.0–8.0)	6.0 (3.0–10.0)	5.0 (3.0–9.0)	
Min–Max	1.0–38.0	1.0–35.0	1.0–38.0	

*N*, number of observations; VS, Verso Vision technology.

* In order to the differences between groups, the variable was categorized as no fall risk (score 0 or 1) and fall risk (score 2, 3, 4 and 5).

Overall, 20 accidental falls were observed, 15 in the group without VS and 5 in the group with VS. The crude incidence rates were 6.65 (95% CI 3.72–10.96) and 2.84 (95% CI 0.92–6.63) per 1,000 person-days in the group without VS and in the group with VS, respectively. The incidence rate ratio was 0.43 (95% CI 0.12–1.24, *p* = 0.0926).

The results of univariable and multivariable Poisson regression models are summarized in Table 3. A statistically significant positive impact of the implementation of VS on the reduction of the number of accidental falls was found in both univariable and multivariable analyses, with a risk reduction of 57% (IRR 0.43, 95% CI 0.25–0.72, *p* = 0.0014) and 79% (IRR 0.21, 95% CI 0.12–0.38, *p* < 0.0001), respectively. Moreover, a lower risk of accidental fall was detected for women (IRR 0.27, 95% CI 0.16–0.47, *p* < 0.0001) among individuals with psychiatric abnormalities (IRR 0.38, 95% CI 0.23–0.62, *p* = 0.0001). Conversely, an age ≥90 years [IRR (vs. <70 years) 2.82, 95% CI 1.39–5.74, *p* = 0.0042], a Stratify score of 2 or higher [IRR (vs. 0) 1.95, 95% CI 1.08–3.53, *p* = 0.0276], a Barthel index between 25 and 65 [IRR (vs. 70–100) 3.06, 95% CI 1.56–5.99, *p* = 0.0011] and an admission due to symptoms, signs, and ill-defined conditions (IRR 4.55, 95% CI 2.73–7.58, *p* < 0.0001) were associated with a higher risk of accidental fall.

**Table 3:** Association between the Verso Vision technology, demographical and clinical characteristics and the number of accidental falls.

Variable	Univariable model				Multivariate model (*N* = 561)	
					Exp (Intercept) (95% CI), *p*-value: 0.005428 (0.002837–0.010388), <0.0001	
	*N*	Exp (Intercept) (95% CI), *p*-value	Incidence rate ratio estimate (95% CI)	*p*-value	Incidence rate ratio estimate (95% CI)	*p*-value
Support of Verso Vision technology (vs. no support)	580	0.006646 (0.005125–0.008619), <0.0001	0.43 (0.25–0.72)	0.0014	0.21 (0.12–0.38)	<0.0001
Female sex (vs. male sex)	580	0.007414 (0.005779–0.009513), <0.0001	0.29 (0.17–0.51)	<0.0001	0.27 (0.16–0.47)	<0.0001
Age (ref. <70 years)	580					
70–89 years		0.004180 (0.002527–0.006915), <0.0001	0.96 (0.53–1.74)	0.8944	0.73 (0.40–1.31)	0.2902
90 years or older			2.53 (1.32–4.84)	0.0052	2.82 (1.39–5.74)	0.0042
Stratify score (ref. score 0)	580					
Score 1		0.004258 (0.002899–0.006253), <0.0001	1.28 (0.73–2.26)	0.3908	1.43 (0.83–2.48)	0.1991
Score 2 or higher			1.29 (0.75–2.23)	0.3548	1.95 (1.08–3.53)	0.0276
Barthel index (ref. 70–100)	572					
0–20		0.003018 (0.001703–0.005349), <0.0001	1.29 (0.64–2.61)	0.4719	1.26 (0.60–2.64)	0.5469
25–65			2.31 (1.20–4.43)	0.0121	3.06 (1.56–5.99)	0.0011
Psychiatric abnormality (vs. absence)	569	0.006518 (0.004855–0.008751), <0.0001	0.57 (0.36–0.91)	0.0193	0.38 (0.23–0.62)	0.0001
Admission for symptoms, signs, and ill-defined conditions (vs. other)	580	0.003894 (0.002983–0.005085), <0.0001	3.66 (2.25–5.96)	<0.0001	4.55 (2.73–7.58)	<0.0001

Incidence rate ratio estimates—univariable and multivariable Poisson regression models. In order to correct estimates for the different length of observation, the logarithmic transformation of the length of the observation was used as offset variable. Deviance was used as scale parameter for the calculation of standard errors. As Poisson regression has the logarithm as the link function these are back transformed results (exponentiated coefficients) of a multiplicative model with incidence rate ratios.

To reduce the bias caused by the non-casual assignment of VS monitoring, sensitivity analysis was carried out weighting each observation with a weight based on the propensity score. A good balance between groups was obtained, with a maximum absolute value of the standardized mean difference equal to 0.02 and the variance ratio ranged between 0.96 and 1.05.

The positive impact of VS monitoring on the reduction of the risk of accidental falls was confirmed at multivariable analysis with a risk reduction of 78% (IRR 0.22, 95% CI 0.13–0.35, *p* < 0.0001).

## 4 Discussion

The epidemiological importance of accidental hospital falls is substantial. Every year, around 1 million patient falls occur in hospitals, resulting in approximately 250,000 injuries and up to 11,000 deaths. About 2% of hospitalized patients fall at least once during their stay. Approximately 25% of falls result in injuries, of which about 10% are serious injuries such as fractures, subdural hematoma, bleeding and death (4, 5). Among all specialties, General Medicine records the highest number of fall reports, followed by Psychiatry and General Surgery (11).

The consequences of accidental falls are of great social and economic importance. Falls during hospitalization lead to a lengthening of hospital stay with a consequent increased risk of exposure to nosocomial infections, need for additional diagnostic and therapeutic activities, increased health and social costs, loss of trust in the health care system, and psychological repercussions, including persistent fear of falling and reduced functional independence (6, 8, 12, 13).

Despite the attention paid by our hospital's management to prevent accidental falls, the possibility to introduce an additional, straightforward system for further reducing the frequency of these events seemed very appealing. Given the scarcity of data in the literature on the efficacy of VS, we felt important to evaluate it with a dedicated study.

Therefore, we designed a study on 362 patients at risk, admitted in 10 rooms with 20 beds, monitored 24 h a day, with a median length of hospitalization of 8 days.

The nursing staff manage the VS system after a brief training period. The implementation of this technology has been straightforward, well-accepted by all operators, and not technically challenging. Patient compliance has been excellent, as the monitoring process does not require any device to be in contact with the body, thereby eliminating discomfort. One limitation of the study design is the retrospective nature of data collection, in which the assignment of the risk group was not randomized. However, to reduce this bias, a sensitivity analysis was performed in which each observation was weighted according to its propensity score. This made it possible to obtain a good balance between groups and therefore to confirm the robustness of results. The findings highlight the efficacy of the VS system. Our analysis demonstrated a marked improvement in patients monitored with VS, showing a 79% reduction in their risk of falls.

In conclusion, this study highlights the ease of use of the VS technology in a hospital setting, with high compliance from both healthcare staff and patients. Furthermore, it suggests that the system has strong potential for effectively reducing and preventing accidental falls in hospitalized patients at risk. Although the presence of false positives and false negatives cannot be entirely ruled out due to the spontaneous nature of the study, none were observed during the observation period. The five patients who experienced falls in monitored beds had been correctly identified by the VS technology as being in precarious conditions and at risk of falling. Alerts were appropriately transmitted to the nursing staff, but for various reasons, they were unable to intervene in time. In contrast, no fall alerts were generated among patients who did not experience any falls. In all cases where the VS generated pre-fall alerts, nurses were able to promptly assist patients in unstable conditions, thereby preventing accidental falls. In order to confirm these encouraging preliminary results, we have prompted the design of a 1-year prospective study, already approved by the Ethics Committee, to assess the efficacy of the VS in a larger series of patients and on a larger number of hospital beds. This study will also enable a comprehensive cost-analysis assessment.

This decision was also supported by the fact that the management of the system by nursing staff, after a short training period, was easy, well accepted by all operators, and without any particular technical problems. Patient compliance was excellent, as monitoring does not involve any device that is in contact with the body, and therefore leaves the patient free of any discomfort.

## Data Availability

The original contributions presented in the study are included in the article/Supplementary Material, further inquiries can be directed to the corresponding author.
